# Silencing the epidermal growth factor receptor gene with RNAi may be developed as a potential therapy for non small cell lung cancer

**DOI:** 10.1186/1479-0556-3-5

**Published:** 2005-06-30

**Authors:** Min Zhang, Xin Zhang, Chun-Xue Bai, Xian-Rang Song, Jie Chen, Lei Gao, Jie Hu, Qun-Ying Hong, Malcolm J West, Ming Q Wei

**Affiliations:** 1 Department of Pulmonary Diseases, Zhong Shan Hospital, Fudan University, Shanghai, PR China; 2 Department of Medicine, University of Queensland, Prince Charles Hospital, Brisbane, Australia

**Keywords:** RNA interference, epidermal growth factor receptor, double stranded RNA, small interference RNA, non small cell lung cancer

## Abstract

Lung cancer has emerged as a leading cause of cancer death in the world. Non-small cell lung cancer (NSCLC) accounts for 75–80% of all lung cancers. Current therapies are ineffective, thus new approaches are needed to improve the therapeutic ratio. Double stranded RNA (dsRNA) -mediated RNA interference (RNAi) has shown promise in gene silencing, the potential of which in developing new methods for the therapy of NSCLC needs to be tested. We report here RNAi induced effective silencing of the epidermal growth factor receptor (EGFR) gene, which is over expressed in NSCLC. NSCLC cell lines A549 and SPC-A1 were transfected with sequence- specific dsRNA as well as various controls. Immune fluorescent labeling and flow cytometry were used to monitor the reduction in the production of EGFR protein. Quantitative reverse-transcriptase PCR was used to detect the level of EGFR mRNA. Cell count, colony assay, scratch assay, MTT assay in vitro and tumor growth assay in athymic nude mice in vivo were used to assess the functional effects of EGFR silencing on tumor cell growth and proliferation. Our data showed transfection of NSCLC cells with dsRNA resulted in sequence specific silencing of *EGFR *with 71.31% and 71.78 % decreases in EGFR protein production and 37.04% and 54.92% in mRNA transcription in A549 and SPC-A1 cells respectively. The decrease in EGFR protein production caused significant growth inhibition, i.e.: reducing the total cell numbers by 85.0% and 78.3 %, and colony forming numbers by 63.3% and 66.8%. These effects greatly retarded the migration of NSCLC cells by more than 80% both at 24 h and at 48 h, and enhanced chemo-sensitivity to cisplatin by four-fold in A549 cells and seven-fold in SPC-A1. Furthermore, dsRNA specific for EGFR inhibited tumor growth *in vivo *both in size by 75.06 % and in weight by 73.08 %. Our data demonstrate a new therapeutic effect of sequence specific suppression of *EGFR *gene expression by RNAi, enabling inhibition of tumor proliferation and growth. However, in vivo use of dsRNA for gene transfer to tumor cells would be limited because dsRNA would be quickly degraded once delivered *in vivo*. We thus tested a new bovine lentiviral vector and showed lentivector-mediated RNAi effects were efficient and specific. Combining RNAi with this gene delivery system may enable us to develop RNAi for silencing EGFR into an effective therapy for NSCLC.

## Background

Lung cancer is a leading cause of cancer death in Australia and the world [1,2]. There are two types of lung cancers, non small cell (NSCLC) and small cell (SCLC). NSCLC accounts for 75–80% of all lung cancers. Overall, NSCLC has a low five-year survival rate of only 8–14%. Furthermore, approximately 75% of all NSCLC patients present with advanced cancers [3]. The goals to manage this group of patients are no longer curative, but instead, palliative, to prolong the survival time through palliation chemotherapy or best supportive care [4]. The median survival of a patient with advanced or metastasis NSCLC is approximately six to eight months [4,5]. Clearly, the future of therapy depends on the development of new 'target agents' that explore methods of inhibiting tumor growth, or sensitizing tumors to chemotherapy or radiation to add to current unsatisfactory therapeutic armament.

Gene therapy is one such strategy being considered. Several genes have been explored, including tumour necrosis factor [6], P53 tumour suppresser gene [7], Herpes Simplex Virus Type-1 (HSV-1) thymidine kinase (TK), and bacterial cytosine deaminase (CD) gene [8]. These approaches generally tried to deliver the gene(s) to cancer cells, and hoped that the transgene would be translated into a protein to provide a therapeutic effect. However, clinical trials have shown that the therapeutic outcome was severely limited by the poor efficiency of current gene transfer vector systems, inadequate weak promoters to drive transgene expression. Therefore, so far, only three cancer gene therapy protocols had reached phase III trials before being terminated.

Epidermal growth factor receptor (EGFR) is a glycoprotein with a molecular weight of 170,000 to 180,000. It is an intrinsic tyrosine-specific protein kinase, which is stimulated upon epidermal growth factor (EGF) binding. The known downstream effectors of EGFR include PI3-K, RAS-RAF-MAPK P44/P42, and protein kinase C signaling pathways. EGFR signaling involved in cell growth, angiogenesis, DNA repair, and autocrine growth regulation in NSCLC as well as in a wide spectrum of human cancer cells [9]. Thus, it has recently emerged as an innovative target for the development of new cancer therapy, particularly for NSCLC [10].

Recently, a monoclonal antibody against EGFR called cetuximab has been developed. It has shown excellent clinical effects for the treatment of lung and head and neck cancer in a clinical trial in humans [11,12]. Other small chemical inhibitors, such as ZD-1839 have also been developed and demonstrated anti-tumor effects in *in vitro *and *in vivo *[13]. However, clinical use of ZD-1839 in humans has not been very successful. Although long term evaluation of the drug is still needed, ZD-1839, as a monoclonal antibody drug (in a protein form), and as with any other drug therapies, was disappointing, demonstrating the need for the development of new and effective technologies [14].

Other novel products based on short DNA and RNA are also currently being developed. These include LY900003 (Affinitac™), OST-774 (Tarceva™), and trastuzumab (Herceptin). LY900003 is an antisense oligonucleotide, known to modify gene expression by interacting with the mRNA involved in the production of disease-specific proteins [15]. However, antisense therapeutics have shortcomings in specificity and consistency.

RNA interference (RNAi) is an evolutionarily conserved process in which recognition of double-stranded RNA (dsRNA) ultimately leads to posttranscriptional suppression of gene expression. This suppression is mediated by short double stranded RNA (dsRNA), also called small interfering RNA (siRNA), which induces specific degradation of mRNA through complementary base pairing. In several model systems, ie.: mostly lower order animals, this natural response has been developed into a powerful tool for the investigation of gene function [16,17]. More recently it was discovered that introducing synthetic 21-nucleotide dsRNA duplexes into mammalian cells could efficiently silence gene expression. Although the precise mechanism is still unclear, RNAi offers a new way to inactivate genes of interest. When compared with traditional antisense knockout technologies, it provides a potential new approach for modulation of oncogenic gene function in cancer cells [18]. In this study, we investigated the possibility whether RNAi could silence EGFR gene in commonly used NSCLC cancer cell lines, A549 and SPC-A1. We also assessed the degree of EGFR gene silencing and its functional outcome in terms of effects on cell proliferation and growth inhibition in vitro and in vivo. Our results suggest that RNAi-mediated silencing of EGFR may provide an opportunity to develop a new treatment strategy for NSCLC.

## Materials and methods

### Cell lines and cell culture

A549 and SPC-A1 are well-characterized human NSCLC cell lines, obtained from the Chinese cell collection facility (Shenergy Biocolor Biological Science & Technology Company, Shanghai, China). Cells were routinely grown in Dulbecco's Modified Eagle's Medium (DMEM, Gibco, USA) supplemented with 10% fetal bovine serum (HyClone, USA) in a humidified atmosphere of 5% CO_2 _at 37°C.

### dsRNA preparation

siRNAs corresponding to EGFR mRNA with dTdT on 3'-overhangs were designed and chemically synthesized according to the recommendation of the manufacturer (Dharmacon Research, USA) [19]. The following sequences were successfully made: siRNA-EGFR sense 5'-GGAGCUGCCCAUGAGAAAUdTdT-3' and antisense 5'-AUUUCUCAUG GGCAGCUCCdTdT-3'. The unrelated nonspecific dsRNAs as control were designed as following: sense 5'-GAACUUCAGGGUCAGCUUG CCdTdT-3' and antisense 5'-GGCAAGCUGACCCUGAAGUUCdTdT-3'. Single strand sense and antisense sequences were annealed into dsRNA following the manufacturer's instructions. The annealed dsRNAs were confirmed on a 15% PAGE gel electrophoresis.

### In vitro transfection

Transfection of dsRNA was performed with commercial reagent, Lipofectamine 2000 (Invitrogen, USA) in 6-well plates following manufacturer's instructions. Briefly, the day before transfection, confluent layers of cells were trypsinized, counted and resuspended. Suspension of 1 × 10^5 ^of cells was plated into each well of the 6-well plates, so that they could become about 70% confluence next day at the time of transfection. Lipofectamine 2000 was diluted in serum-free DMEM and mixed with dsRNA at a 1:2 ratio (4 μl of 20 μmol/L of siRNA formulated with 8 μl of Lipofectamine 2000). The formulation of the mixture continued at room temperature and was applied 25 min later in a final volume of 2 ml per well. The cells were then incubated for another 48 h. Cell numbers were determined using a hemocytometer before subsequent assays.

### Assessment of the EGFR numbers

The numbers of EGFR in both cell lines were determined by an immuno-fluorescent assay as previously reported [20]. The cells were harvested by trypsinization, washed twice with 1 × PBS, and incubated with mouse anti-EGFR monoclonal antibodies (mAb), (generously donated by the Shanghai Institute of Cell Biology, Chinese Academy of Sciences) for 1 h at 37 °C. The cells were then washed and stained with FITC-conjugated rabbit anti-mouse antibody (Antibody Diagnostic Inc., Shanghai) and left for incubation in the dark. After 45 min, the cells were subsquently washed twice and fixed in 0.5 ml of 4% para-formaldehyde. The stained cells were analyzed by a fluorescent microscopy or on a Becton Dickinson FACScan with excitation and emission settings at 488 nm and 530 nm respectively. The numbers of EGFR on NSCLC cells were finally calculated as the percentage of positive cells × mean intensity of fluorescence.

### RNA isolation and complementary DNA synthesis

Total RNA was extracted from cell pellets using Trizol reagent following manufacturer's instructions (Gibco BRL, Canada) and dissolved in TE buffer. Total RNA was quantified with a spectrophotometer (Pharmacia Biotech, Piscataway, NJ). To get rid of possible contamination by genomic DNA, total RNA was treated with DNase I (Invitrogen, USA) for 15 min at room temperature. The reaction was stopped by addition of 25 mM EDTA and heated at 65°C for 10 min followed by 95°C for 5 min. For complementary DNA (cDNA) synthesis, 400 ng of total RNA was transcribed with cDNA transcription reagents using 0.8 μg of the oligo(dT)_18 _primer for subsequent quantitative, real-time polymerase chain reaction (PCR).

### Quantitative Reverse-Transcriptase PCR

This was performed using an ABI Prism 7700 sequence detection system (Applied Biosystems) as described previously [21]. Primers and TaqMan probes were designed using the Primer Express TM 1.0 (Applied Biosystems) software to amplify approximately 150 base pairs of sequences. Probes were labeled at 5' end with the reporter dye molecule FAM (6-carboxy-fluorescein) and at 3' end with quencher dye molecule TAMARA (6-carboxytetramethyl-rhodamine). Real-time PCRs were conducted in a total volume of 50 μl with 1 × TaqMan Master Mix (Applied Biosystems) and primers at 300 nM and probes at 200 nM. Primer sequences were as follows: EGFR gene forward primer, 5' -CGAGGGCAAATACAGCTTTG -3'; backward primer, 5'- CCTTCGCACTTCTTACACTTG -3'; probe 5'FAM-ACGCCGTCTTCCTCCATCTCATA GC-TAMRA3'. Thermal cycler parameters included one cycle at 94°C for 2 min, and 45 cycles involving denaturation at 94°C for 10 s annealing at 53°C for 30 s and extension at 72°C for 40 s, followed by a final extension at 72°C for 10 min. The relative amount of EGFR cDNA in each sample was calculated by dividing the C_T _value with the corresponding value of the housekeeping gene glyceraldehyde-3-phosphate dehydrogenase (GAPDH). Negative controls were included in each experiment to ensure the reagents were free of contamination.

### Colony forming assay

The number of colonies was determined as described previously [21]. Briefly, after transfection with dsRNA and various controls, cells were trypsinized, counted, and seeded for the colony forming assay in 60 mm dishes at 300 cells per dish. After incubation for 14 days, colonies were stained with crystal violet and the numbers of positive cells counted. Colonies containing more than 50 cells were scored, and triplicates containing 10–150 colonies/dish were counted in each treatment.

### Scratch assay

This was performed as previously described [22]. Cells were seeded in triplicate on collagen IV coated 60 mm tissue culture dishes at 1 × 10^5^cells/dish. A scratch through the central axis of the plate was gently made using a pipette tip 4 h after the cells were transfected with specific dsRNA or various controls. Migration of the cells into the scratch was observed at two separate time points of 24 h and 48 h.

### Chemo-sensitivity assay

This was performed as described previously [23]. Briefly, after transfection with specific dsRNA or various controls in 6-well plates for 24 h, cells were trypsinized, and seeded into 96 well plates. Cells were, after overnight culture, exposed to increasing concentration of cisplatin ranged from 0 to 50 ng/ml for another 24 h. MTT of 20 μl (1 mg/ml) was added to each well for 4 h at 37°C to allow MTT to form formazan crystals by reacting with metabolically active cells. Subsequently the formazan crystals were solubilized by 150 μl of DMSO. The absorbance of each well was measured in a microplate reader at 490 nm (A_490_). The percentage of cell growth was calculated by comparison of the A_490 _reading from specific dsRNA transfected cells versus control transfected cells.

### Inhibition of tumor growth in athymic nude mice

Athymic nude mice (3–4 week old male) were obtained from Shanghai Institute of Cell Biology, Chinese Academy of Sciences, maintained under aseptic conditions and cared in accordance with institutional guidelines. They were then randomized into five groups with 6 mice per group: ie.: negative control, positive control, transfection reagent control, unrelated dsRNA control, and dsRNA specific for EGFR group (dsRNA-EGFR). After plating into 10 cm plate and transfection in vitro, SPC-A1 cells (~1 × 10^6^) in 50 μl of PBS were injected *s.c*. into the left flank area of the mice. An equal volume of PBS was injected into negative control group. Tumor volumes were determined by direct measurement with calipers and calculated using the formulaπ/6 × (larger diameter) × (small diameter). Human tumor xenografts were allowed to grow to a size of 10 mm × 10 mm before the mice were sacrificed and tumors removed and weighed.

### Statistical analysis

The silencing effects of dsRNA on *EGFR *on cell growth, colony formation, cell migration and tumor growth *in vivo *were analyzed by student *t *test. Differences were considered to be significant at P < 0.05. SPSS10.0 software was used to perform statistical analysis. In the experiments for testing chemo-sensitivity that involved multiple cisplatin doses, the linear quadratic model was fitted with Origin 6.0 software.

### ResultsSignificant down-regulation of EGFR gene expression with siRNA specific for EGFR

**Figure 1:**
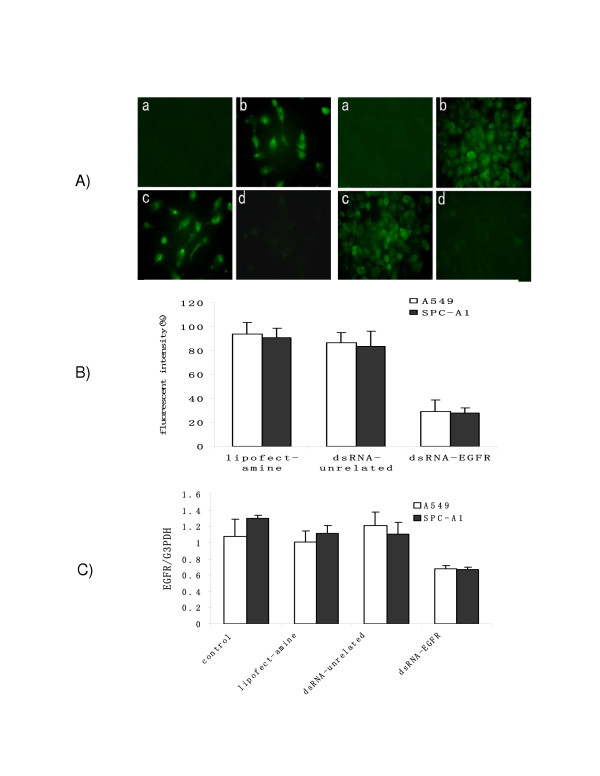
SiRNA-mediated RNAi effects in A549 and SPC-A1 cells. (A) dsRNA-mediated inhibition of EGFR gene expression in A549 cells (upper) and SPC-A1 cells (lower). Fluorescence images taken 48 h after transfection were shown. (a) Cells were stained with FITC-conjugated secondary antibody. (b) Cells were stained with an EGFR-specific antibody and secondary antibody. (c) FITC staining of cells transfected with unrelated dsRNA. (d) FITC staining of cells transfected with dsRNA-EGFR. (B) EGFR gene expression was quantified in both control and transfected cells by a flow cytometry with excitation and emission settings of 488 nm and 530 nm respectively. Cells were transfected for 48 h, then stained with an EGFR-specific antibody. Results were expressed as the percentage of fluorescent intensity relative to controls. Each column represented the mean of three replicated experiments. (C) EGFR gene level was quantified by real-time PCR. Expression of EGFR mRNA was analyzed using a semiquantitative real-time PCR assay. The relative gene levels were calculated in relation to the expression of the housekeeping GAPDH gene.

Single stranded synthetic RNAs were firstly annealed together to form dsRNA and then transiently transfected into tumour cell lines. Forty-eight hours after transfection, the expression of the EGFR was examined. The fluorescent immune labeling assay demonstrated that the number of the EGFR on the cell membrane was significantly and specifically inhibited by the transfection of dsRNA specific for EGFR, but not by unrelated dsRNA (Fig. 1A c–d), nor by the transfection reagent control and negative control (without an mAb, Fig. 1A a–b). The number of the EGFR assessed by a flow cytometry was also in agreement with the immune assay in which dsRNA-EGFR dramatically reduced EGFR gene expression to levels that were 71.31% and 71.78% less than those seen in control groups (P < 0.001). There were no differences in the intensity of fluorescence in the control groups, i.e.: both of the transfection reagent control as well as unrelated dsRNA group, did not show any significant decrease (P > 0.05) (Fig. 1B &1C), suggesting the reduction of the EGFR protein was significant and specific.

Further molecular analyses revealed that the down-regulation of EGFR expression was the result of a marked decrease in the transcriptional activity of *EGFR*. As shown in Fig. 1E, EGFR specific mRNA were strongly down-regulated by 37.04% and 54.92% in A549 and SPA-A1 cells treated with dsRNA-EGFR (P < 0.01). As anticipated, mRNA transcription was not significantly inhibited in groups (P > 0.05).

### DsRNA specific for EGFR also inhibited tumor cell growth

**Figure 2:**
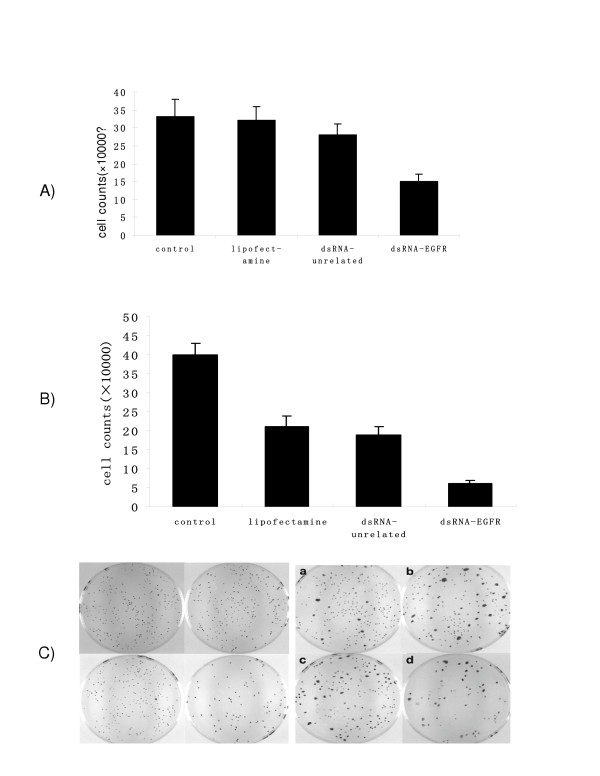
The effects of dsRNA-EGFR on A549 (A) and SPC-A1 (B) cell count and colony formation (C). Cells were seeded into 6 well plates at a density of 1 × 10^5 ^per well. Forty-eight hours after transfection, cell numbers were determined using a hemocytometer. For colony forming assay, cells transfected with siRNA,

To investigate the functional effect of the down regulation of the EGFR expression, we performed two experiments; one was a cell count assay and another, a colony forming assay. Cell count results showed a significant decrease in the number of cells by 85.0% in A549 and 78.3% in SPC-A1 (P < 0.001) (Fig 2A) when transfected with dsRNA-EGFR. In comparison, the number of cells in the control groups was high and consistent in both cell types.

The results of the colony forming assay were in agreement with those of cell count. A significant decrease in the number of colonies by 63.3% in A549 and 66.8% in SPC-A1 was apparent when the cells were transfected with dsRNA-EGFR. In contrast, cells in the control groups showed little decrease in the number of colonies. These results suggest the transfection of NSCLC cells with dsRNA-EGFR caused dramatic growth inhibition of the tumor cells (P < 0.001) (Fig 2B).

### DsRNA specific for EGFR retarded the migration of NSCLC

**Figure 3:**
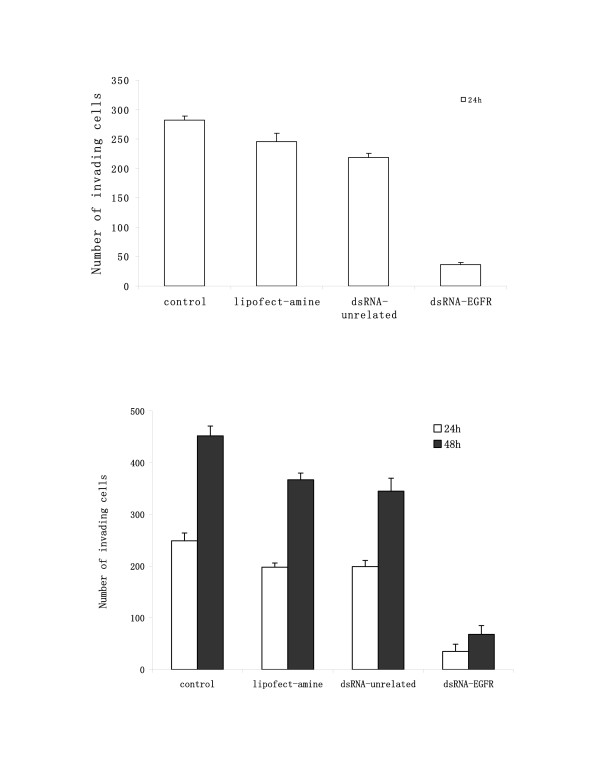
Effects of siRNA-EGFR on NSCLC cells' ability to migrate. NSCLC cells in control group showed higher motility in a standard scratch assay. The migration of A549 cells (upper) and SPC-A1 cells (lower) was quantitatively assessed at time points of 24 h (white) and 48 h (black) after the introduction of a scratch in monolayer transfected cells grown on collagen IV. Each column represented the mean of three experiments; bars, SD.

To determine whether gene silencing affected the ability of A549 and SPC-A1 cells to migrate, a scratch assay was performed by introduction of a scratch on the monolayer of cells grown on collagen IV coated plates. The results were quantitatively assessed at 24 h and 48 h and showed that NSCLC cells transfected with dsRNA-EGFR had a very low motility at both time points, representing a retarded migration by more than 80% (Fig 3) (P < 0.01). The results showed that dsRNA-EGFR transfected cells had almost lost the ability to migrate, thus suggesting that siRNA had the potential to reduce the invasiveness of NSCLC, thus blocking migration and metastasis of NSCLC tumours.

### dsRNA sensitized NSCLC cell to chemo-therapeutic agent cisplatin

**Figure 4:**
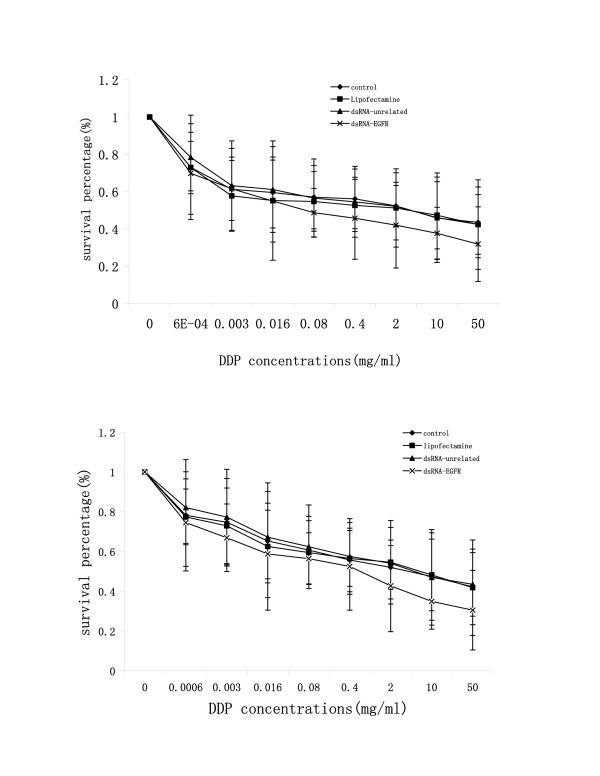
Effect of dsRNA-EGFR on chemosensitivity of NSCLC cells to cisplatin. Cells were transfected with siRNA, then exposed to various doses of cisplatin for 48 h and viability was accessed. The percentage of cell growth was calculated by comparison of the A_490 _reading from treated versus control cells. The IC_50 _value of A549 (upper) cells to cisplatin in control group, transfection reagent control, unrelated dsRNA group and dsRNA-EGFR group was 2.67 μg/ml, 2.30 μg/ml, 2.19 μg/ml and 0.50 μg/ml respectively. The IC_50 _value of SPC-A1 cells to cisplatin (lower) was 3.67 μg/ml, 3.11 μg/ml, 3.07 μg/ml and 0.43 μg/ml respectively.

To test what effect RNAi would have on the sensitivity of NSCLC cells to a chemotherapeutic agent, we designed an experiment to examine the changes of sensitivity to cisplatin before and after transfection with dsRNA. Cisplatin is a commonly used chemical agent clinically. The experiment was performed by examining cells' viability using a MTT assay. As shown in Fig. 4, we demonstrated that cells transfected with dsRNA-EGFR were more sensitive to cisplatin than various control groups. More importantly, this sensitizating effect was dose-dependent, showing a significant correlation of growth inhibition with doses of cisplatin used. When the data was further analyzed based on the value of IC_50 _using Origin 6.0 software, we demonstrated that transfection of dsRNA-EGFR increased the sensitivity of A549 and SPC-A1 to cisplatin by four- and seven-fold respectively.

### DsRNA specific for EGFR inhibited tumor growth in vivo

**Figure 5:**
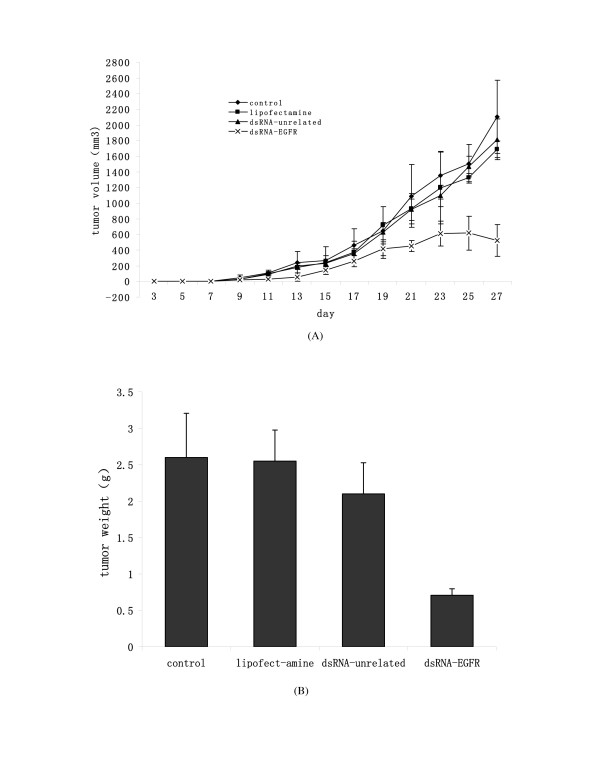
Inhibition of tumor growth in vivo by dsRNA specific for EGFR. Transfected SPC-A1 cells (~1 × 10^6^) were injected into the left flank area of the mice and tumor xenografts were allowed to grow to a size of approximately 10 mm × 10 mm. After mice were sacrificed, tumors were removed and weighed. (A) Tumor volumes measured by calipers every 2 days. (B) Tumor weight. Each column represented the mean of six mice, bars, SD.

Clearly, it would be more relevant to the development of a therapeutic protocol if we could prove that transfection of siRNA could cause growth inhibition *in vivo*. In this experiment, we investigated the possibility using a mouse model of human tumor xenograft. Human SPC-A1 cells were selected for the in vivo experiment because its subcutaneous xenografts formed quicker than A549 (Zhang et al., unpublished data). Approximately 1 × 10^6 ^of SPC-A1 cells were transfected with dsRNA specific for EGFR or various controls, and then injected into the left flank area of the mice. Tumor growth was monitored and xenografts were allowed to grow to a size of approximately 10 mm × 10 mm. After mice were sacrificed, tumors were removed and weighed. As shown in Fig. 5, the time when tumors were first visible in control groups was 6–8 days earlier than that in dsRNA-EGFR transfected group. Tumors in mock control group, transfection reagent control and unrelated dsRNA transfected control groups were of a similar size, all of which had a much larger solid tumor and reddish appearance, whereas those from dsRNA-EGFR transfected group remained small and pale. When mice were sacrificed and the size of the tumors compared, tumors from the dsRNA-EGFR transfected group showed significant smaller size of 75.06 % and weight of 73.08% (P < 0.01), demonstrating an in vivo growth inhibitory effect.

### Development of a uniqe lentiviral vector system encoding dsRNA

No doubt, the in vivo use of dsRNA for gene transfer to tumor cells would be limited because dsRNA would be quickly degraded once delivered *in vivo*. In addition, the efficiency of dsRNA-mediated gene transfer into tissues *in vivo *would be very low and thus very limited. To improve gene delivery to a variety of cancer cells *in vivo*, we developed a new bovine lentiviral vector (Jembrana Disease Virus, JDV)-mediated delivery of RNAi approach. The JDV vector system was recently developed in our laboratory and has shown efficient gene delivery to a variety of cell types both in dividing and non- dividing cycles [24,25].

Based on the JDV backbone, two 56 bp DNA fragments that contain 19 bp of the EGFP in sense and anti-sense orientation with a hairpin liker were synthesized, annealled into double strands and cloned in a cassettes down stream of a polymerase-III H1- RNA promoter (PIII). This promoter directs transcription of the dsDNA into dsRNA with well-defined start site with a termination signal consisting of five thymidines in a row. Most importantly, the cleavage of the dsRNA transcript at the termination site is after the second uridine, yielding a transcript resembling the end of synthetic dsRNA.

The new JDV vector encoding the dsRNA specific for EGFR is named pjLPIIIRE. Introduction of pjLPIIIRE together with the packaging construct, pjPack and the envelop construct, pCMV-VSVG into the 293T cells produced VSV-G pseudotyped JDV vectors. The vector harvests were further concentrated and titrated as we previously reported [24,25]. These vectors were used to transducer A549 and SPC-A1 cells. At a multiplicity of infection (MOI) of 10 and 72 hours after transduction, RNAi-mediated gene silencing of EGFR was apparent. About 75% of A549 and 80% of SPC-A1 cells became EGFR negative, demonstrating an effective RNAi-mediated silencing of the EGFR gene. Control constructs of JDV vectors encoding a dsRNA specific for the enhanced green fluorescent protein (EGFP) did not show a significant inhibitory effect at a higher concentration of the vector (MOI of 15). However, in cells that were previously transduced with EGFP marker gene and expressing the EGFP protein in approximately 100% of the cells, about 75% of A549 and 80% of SPC-A1 cells became EGFR negative, a results similar to EGFR silencing. These results suggested lentivector-mediated RNAi effects were efficient and specific and would be a useful tool for in vivo gene delivery for the therapy.

## Discussion

Increased EGFR expression is common in various cancers. This has correlated with neoplastic progression of these cancers. Blockade of this oncogenic EGFR signaling pathway may represent a promising strategy for the development of selective anticancer approaches. However, although considerable progress has been made in the development of EGFR-targeted antibodies or small molecule tyrosine kinase inhibitors, data from the current clinical trials showed that there were some shortcomings with these approaches, namely ineffective in some cases and emergence of resistance in others [14]. In an effort to develop new strategies to block the over expression of EGFR in NSCLC cells, we examined the potential of a dsRNA-mediated specific RNAi approach for silencing the EGFR in NSCLC cell lines in vitro and in vivo. We demonstrated a significant inhibition of EGFR gene expression in both A549 and SPC-A1 cells. RNAi dramatically reduced the EGFR transcription and down regulated protein production. This, in turn, translated into a range of growth inhibitory effects on the tumor cells *in vitro *and *in vivo*, including a significant tumor growth delay and impairment in xenograft animal models. Our results are the first that show a significant silencing of an endogenous cellular gene and growth inhibition *in vivo*.

Current data supports the notion that in mammalian cells, RNAi is superior to antisense approaches for down regulation of gene expression though antisense had been widely used [26]. Furthermore, dsRNA was more sensitive, specific and stable than antisense RNA or DNA partly due to its dTdT 3'-overhangs on each strand [27]. We selected the 21-nt sense and antisense ssRNAs targeting against EGFR from the coding regions and added dTdT 3'-overhangs on each strand to increase siRNA stability. Our current results were in agreement with reported data that capped dsRNA was active and stable [28]. The RNAi effects that we achieved more than doubled the effects of antisense that was normally around 30% of down regulation of gene expression (unpublished data).

It had been suggested that the degree to which the expression of a given protein to be inhibited through RNAi depended on the half-life and its synthesis rate of that protein. Due to its much faster turnover, EGFR was considered to be more difficult to knock down using RNAi. This was because even if the existing ready-made protein was blocked, previously synthesized molecules persisted in the plasma membrane for a relatively long time [29]. Nevertheless the fact that we successfully inhibited the expression of EGFR as well as the function of EGFR in NSCLC cells provided convincing evidence that RNAi is a very potent technique. To our knowledge, these results represent the first demonstration that chemically synthetic 21-nt siRNA was readily delivered into NSCLC cell lines and effectively suppressed the oncogenic gene expression and function.

Experimental and clinical data had indicated that EGFR over expression correlated with various critical processes in the development, maintenance, and spread of malignant tumors.[30] The reduction of EGFR may lead to a failure in downstream signal cascades including PI3-K, RAS-RAF-MAPK P44/P42, and protein kinase C pathway, and subsequently block the routes to activation of more direct modulators of mitogenesis and other cancer-promoting phenotypes [13]. Initially, due to the general belief that RNAi only induces a gene knock down, rather than a complete knock out, we were not quite sure what functional outcome could be achieved by knocking down EGFR, if any. Our results were most surprising and significant in that even though EGFR transcription was only reduced by about 50% and protein production by 70%, which means that the transfected cells would still express more than 20% of receptors, these cells displayed, however, dramatic growth inhibition in the number of cell count, and colony formation and significantly retarded cell growth. These results convincingly suggested that even an incomplete suppression of EGFR expression was sufficient to hinder growth factor-mediated signaling. This was consistent with the hypothesis that the activation of the tyrosine phosphorylation response might be completely switched off if the number of EGFR dropped below a given threshold [31]. This notion is extremely important for the development of a RNAi-mediated therapy of NSCLC because a complete gene inactivation in these cells, which is difficult to achieve is no longer needed suggesting RNAi based gene silencing may be developed into a therapy that could blunt the invasiveness of cancer cells, even stop metastasis.

Up till now, the effects of EGFR on chemo-sensitivity were less clear and were suggested that it might be depending on particular tumor cell lines or cell types or particular drugs used for the experiment. However, a growing number of reports had supported the idea that activation of the EGFR signal transduction pathway might induce chemo-resistance in NSCLC cells [32]. This might provide a rationale to down regulate EGFR, thus improving chemosensitivity. One of the commonly used drugs was cisplatin, a DNA-damaging anticancer agent. It has been demonstrated that cisplatin modified DNA repair activity in a variety of different experimental systems [33]. Our study found that the reduction of EGFR resulted in enhanced chemo-sensitivity to cisplatin by four-fold in A549 cells and by seven-fold in SPC-A1 cells. In previous studies using different receptor inhibitors, the degree of EGFR-induced enhancement of sensitivity to cisplatin was only in the range of 2- to 4- fold. Our seven-fold enhancement in SPC-A1 cells suggested that RNAi caused better suppression of the EGFR and increased significantly more chemo-sensitivities than the previous approaches. This would represent a clinical significance if translated into clinical setting because cisplatin resistance could be potentially overcome by this approach [34]. The chemo-sensitization of NSCLC cells with dsRNA-EGFR indicated that dsRNA-EGFR is a more fascinating candidate for further development into a better therapy. Clearly, for the development of such a therapeutic strategy for clinical use, a suitable vector system is necessary. We have showed the new lentiviral vector system was capable of delivery dsRNA and caused a significant silencing effect. Our development of the bovine lentiviral vector system for the delivery of dsRNA would benefit the use of the technology in vivo for NSCLC. Hopefully in the near future, strategies based on RNAi will be ready for preclinical or clinical trials.

## References

[B1] McLennan G., Roder DM (1989). Lung cancer in Australia. Med J Aust.

[B2] Burton RC (2002). Cancer control in Australia: into the 21 (st) Century. Jpn J Clin Oncol.

[B3] Hansen HH (2002). Treatment of advanced non-small cell lung cancer. BMJ.

[B4] Cridelli C (2004). Targeted therapies in the treatment of non small cell lung cancer:. Curr Opin Oncol.

[B5] Evans TL, Lynch TJ (2001). Lung Cancer. Oncologist.

[B6] Weichselbaum RR, Hallahan DE, Beckett MA (1994). Gene therapy targeted by radiation preferentially radiosensitizes tumor cells. Cancer Res.

[B7] Gallardo D, Drazan KE, Mcbride WH (1996). Adenovirus-based transfer of wild-type p53 gene increases ovarian tumor radiosensitivity. Cancer Res.

[B8] Rogulski KR, Kim JH, Kim SH (1997). Glioma cells transduced with an Escherichia coli CD/HSV-1 TK fusion gene exhibit enhanced metabolic suicide and radiosensitivity. Hum Gene Ther.

[B9] Wakeling AE (2002). Epidermal growth factor receptor tyrosine kinase inhibitors. Curr Opin Pharmacol.

[B10] Wakeling AE, Guy SP, Woodburn JR (2002). ZD1839 (Iressa): an orally active inhibitor of epidermal growth factor signaling with potential for cancer therapy. Cancer Res.

[B11] Shin DM, Donato NJ, Perez-Soler R (2001). Epidermal growth factor receptor-targeted therapy with C225 and cisplatin in patients with head and neck cancer. Clin Cancer Res.

[B12] Ciardiello F, Caputo R, Blanco R (2000). Inhibition of growth factor production and angiogenesis in human cancer cells by ZD-1839 (Iressa), a selective epidermal growth factor receptor tyrosine kinase inhibitor. Clin Cancer Res.

[B13] Shawver LK, Slamon D, Ulrich A (2002). Smart drugs: tyrosine kinase inhibitors in cancer therapy. Cancer Cell.

[B14] Baselga J (2001). The EGFR as a target for anticancer therapy – focus on cetuximab. Eur J Cancer.

[B15] Tamm I, Dorken B, Hartmann G (2001). Antisense therapy in oncology, new hope for an old idea?. Lancet.

[B16] Elbashir SM, Lendechel W, Tuschl T (2001). RNA interference is mediated by 21- and 22-nucleotide RNAs. Genes Dev.

[B17] Hammond SM, Caudy AA, Hannon GJ (2001). Post transcriptional gene silencing by double stranded RNA. Nat Rev Genet.

[B18] Elbashir SM, Harborth J, Lendechel W (2001). Duplexes of 21-nucleotides RNAs mediate RNA interference in cultured mammalian cells. Nature.

[B19] Christen RD, Hom DK, Porter DC (1990). Epidermal growth factor regulates the in vitro sensitivity of human ovarian carcinoma cells to cisplatin. J Clin Invest.

[B20] Kroning R, Jones JA, Hom DK (1995). Enhancement of drug sensitivity of human malignancies by epidermal growth factor. Br J Cancer.

[B21] Zhang M, Zhang X, Bai CX, Chen J, Wei MQ (2004). Inhibition of epidermal growth factor receptor (EGFR) by RNA interference in A549 cells. Acta Pharmacol Sin.

[B22] Lal A, Glazer CA, Martinson HM (2002). Mutant epidermal growth factor receptor up-regulates molecular effectors of tumor invasion. Cancer Res.

[B23] Liu CS, Kong BH, Xia HQ (2001). VP22 enhanced intercellular trafficking of HSV thymidine kinase reduced the level of ganciclovir needed to cause suicide cell death. J Gene Med.

[B24] Metharom P, Tayra S, Xia HQ, MacMillian J, Shepherd R, Wilcox G, Wei MQ (2002). New bovine lentiviral vectors based on the Jembrana disease virus. J Gene Medicine.

[B25] Zhang B, Xia HQ, Cleghorn G, Gobe G, West M, Wei MQ (2001). A highly efficient and consistent method for harvesting large volumes of high titre lentiviral vectors. Gene Therapy.

[B26] Nagy P, Arndt-Jovin DJ, Jovin TM (2003). Small interfering RNAs suppress the expression of endogenous and GFP-fused epidermal growth factor receptor (erbB1) and induce apoptosis in erbB1-overexpressing cells. Exp Cell Res.

[B27] Nieth C, Priebsch A, Stege A (2003). Modulation of the classical multidrug resistance (MDR) phenotype by RNA interference(RNAi). FEBS Lett.

[B28] Tsai CM, Chang KT, Li L, Perng RP, Yang LY (2000). Interrelationships between cellular nucleotide excision repair, cisplatin cytotoxicity, HER-2/neu gene expression and epidermal growth factor receptor level in non-small cell lung cancer. Jpn J Cancer Res.

[B29] Somasundaram K (2003). Stable RNA interference (RNAi) in mammalian cells. Cancer Biol Ther.

[B30] Magne N, Fischel JL, Dubreuil A (2002). Sequence-dependent effects of ZD1839 ('Iressa') in combination with cytotoxic treatment in human head and neck cancer. Br J Cancer.

[B31] Hirata A, Ogawa S, Kometani T (2002). ZD1839 (Iressa) induces antiangiogenic effects through inhibition of epidermal growth factor receptor tyrosine kinase 2. Cancer Res.

[B32] Nakagawa K (2001). Tyrosine kinase inhibitors-solid cancers. Gan To Kagaku Ryoho.

[B33] Huang SM, Li J, Armstrong EA (2002). Modulation of radiation response and tumor induced angiogenesis after epidermal growth factor receptor inhibition by ZD1839 (Iressa). Cancer Res.

[B34] Williams KJ, Telfer BA, Stratford IJ (2002). ZD1839 ('Iressa'), a specific oral epidermal growth factor receptor-tyrosine kinase inhibitor, potentiates radiotherapy in a human colorectal cancer xenegraft model. Br J Cancer.

